# Resistant starch type-4 intake alters circulating bile acids in human subjects

**DOI:** 10.3389/fnut.2022.930414

**Published:** 2022-10-20

**Authors:** Samitinjaya Dhakal, Moul Dey

**Affiliations:** School of Health and Consumer Sciences, South Dakota State University, Brookings, SD, United States

**Keywords:** circulating bile acid, resistant starch type 4, metabolic syndrome, microbiota, dietary fiber, metabolomics

## Abstract

**Background:**

Resistant starch (RS) type 4 (RS4) is a type of RS, a class of non-digestible prebiotic dietary fibers with a range of demonstrated metabolic health benefits to the host. On the other hand, bile acids (BA) have recently emerged as an important class of metabolic function mediators that involve host-microbiota interactions. RS consumption alters fecal and cecal BA in humans and rodents, respectively. The effect of RS intake on circulating BA concentrations remains unexplored in humans.

**Methods and results:**

Using available plasma and stool samples from our previously reported double-blind, controlled, 2-arm crossover nutrition intervention trial (Clinicaltrials.gov: NCT01887964), a liquid-chromatography/mass-spectrometry-based targeted multiple reaction monitoring, and absolute quantifications, we assessed BA changes after 12 weeks of an average 12 g/day RS4-intake. Stool BA concentrations were lower post RS4 compared to the control, the two groups consuming similar macronutrients (*n* = 14/group). Partial least squares-discriminant analysis revealed distinct BA signatures in stool and plasma post interventions. The increased circulating BA concentrations were further investigated using linear mixed-effect modeling that controlled for potential confounders. A higher plasma abundance of several BA species post RS4 was observed (fold increase compared to control in parenthesis): taurocholic acid (1.92), taurodeoxycholic acid (1.60), glycochenodeoxycholic acid (1.58), glycodeoxycholic acid (1.79), and deoxycholic acid (1.77) (all, *p* < 0.05). Distinct microbiome ortholog-signatures were observed between RS4 and control groups (95% CI), derived using the Piphillin function-prediction algorithm and principal component analysis (PCA) of pre-existing 16S rRNA gene sequences. Association of *Bifidobacterium adolescentis* with secondary BA such as, deoxycholic acid (rho = 0.55, *p* = 0.05), glycodeoxycholic acid (rho = 0.65, *p* = 0.02), and taurodeoxycholic acid (rho = 0.56, *p* = 0.04) were observed in the RS4-group, but not in the control group (all, *p* > 0.05).

**Conclusion:**

Our observations indicate a previously unknown in humans- RS4-associated systemic alteration of microbiota-derived secondary BA. Follow-up investigations of BA biosynthesis in the context of RS4 may provide molecular targets to understand and manipulate microbiome-host interactions.

## Introduction

Bile acids (BA) are a family of steroid molecules synthesized from cholesterol in hepatocytes, stored in the gallbladder, and are released into the intestine to assist the absorption of dietary fats and vitamins. BA also function as signaling molecules through the membrane G protein-coupled receptor 5 and the Farnesoid X receptor to influence various metabolic processes and energy homeostasis (1, 2). The BA pool consists of primary and secondary BA species as well as their glycine and taurine conjugates (2). In humans, cholic acid and chenodeoxycholic acid are the two primary BA species. They are produced in the liver utilizing two biosynthetic pathways. The main BA pathway is initiated by cholesterol 7α-hydrolase (CYP7A1) to produce cholic acid and chenodeoxycholic acid, and the alternative pathway is initiated by sterol-27-hydroxylase to produce chenodeoxycholic acid. The main pathway is the major source of both primary BA species (1, 3). Most BA are then conjugated with glycine or taurine in the hepatocytes with the enzymes such as bile acid: CoA synthase and bile acid: amino transferase (2, 3). Gut microbiota metabolizes the conjugated or unconjugated primary BA into secondary BA—deoxycholic acid, lithocholic acid, and ursodeoxycholic acid—through deconjugation, dehydroxylation, and dehydrogenation in the colon (4). Most secondary BA are reabsorbed in the brush border membrane of the intestine into portal blood circulation, followed by glycine and/or taurine conjugation in the liver before being stored in the gall bladder. Microbial involvement results in increased diversity and hydrophobicity of the BA pool, thus, altering the rate of intestinal reabsorption and fecal elimination of BA (4).

Dietary modulation of the gut microbiota may influence circulating BA with potential downstream effects on the host (4–7). We and others have shown that resistant starches (RS) represent fermentable carbohydrates with a range of potential health benefits (8–10). RS are also naturally low in calories due to escaping enzymatic digestion in the small intestine (11–13). Physicochemical properties and physiological impact on the host may vary by RS type (RS1, RS2, RS3, and RS4) (14). It is also possible that the differences in the chemical structures among RS determined their variable accessibility to different gut microbes (9, 10, 13, 15, 16), potentially resulting in distinct modulation of microbial metabolites. In this context, human studies reporting the effects of RS on BA are a handful: van Munster et al. reported increased primary BA in feces after RS2 intake (17). Langkilde et al. observed decreased total BA in ileostomy excreta post RS3 consumption, while Grubben et al. reported lower total and secondary BA concentrations in fecal water after feeding RS2 (18, 19). In contrast, animal model studies have demonstrated a range of alterations related to the fecal and cecal BA in response to RS2, RS3, or RS4 intake (16, 20–27). More recently, Bindels et al. observed that RS2 and RS4 could alleviate Western-type diet-induced cecal BA signature in mice (16). However, the effect of any RS on circulating BA is less clear, with only one mouse study observing an RS2-induced increase in circulating primary BA and deoxycholic acid (28). We are not aware of any human subject or animal-model studies reporting RS4-induced circulating BA signature.

Phosphorylated cross-linked RS4 produced from wheat starch is marketed as a dietary fiber that can be used as a stealth ingredient to swap higher-calorie starches without compromising palatability or major recipe alterations (29–31). We and others have shown that a diet supplemented with this food-ingredient may attenuate blood glucose and cholesterols, increase satiety, and lower body weight, fat, and inflammation (8, 16, 29, 31, 32). A similar RS4 product derived from maize has also demonstrated metabolic health benefits (33, 34). This study leveraged available stool and plasma samples from our previously reported double-blind controlled two-arm crossover trial of RS4 intervention completed over 26 weeks involving Hutterite participants living in the upper Midwestern US (29). Hutterites represent a culturally unique and homogenous minority group of central European ancestry, practicing a communal lifestyle. During the active trial period, the participants consumed ∼12 g/day of RS4 or regular wheat starch as control, each for 12 weeks without any gastrointestinal side effects. Previously, we have reported the impact of RS4 consumption on anthropometric characteristics, biomarkers of inflammation, lipid and glucose metabolism, microbiota composition and diversity, and short-chain fatty acids (8, 29). Here we examined the effects of RS4 intake on individual BA species and microbiota functional capacity in adults with metabolic syndrome. The effect of RS4 on human fecal and circulating BA remains poorly understood.

## Materials and methods

### Clinical trial overview

**FIGURE 1:**
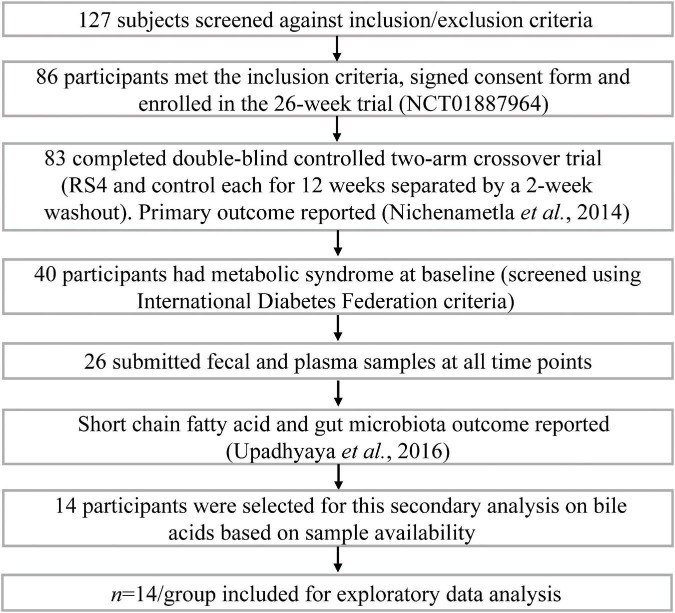
Participant flow in the study.

The parent clinical trial from which this study originates was registered as NCT01887964 at clinicaltrials.gov. The trial was approved by the Institutional Review Board for Human Subjects Research at South Dakota State University (1112012-CR) and followed the Declaration of Helsinki. Details about the trial, primary outcomes, and microbiome output have been previously reported (8, 29). Briefly, the trial was conducted in two Hutterite colonies in eastern South Dakota, USA, as a double-blind (participants and investigators), controlled, two-arm crossover study (Figure 1). Each arm of the intervention was 12-weeks long separated by a 2-weeks washout period. The sequence of the intervention was cluster-randomized due to community living and dining practices: one colony received control wheat flour first, while the other colony received RS4 flour first, and then were crossed over. RS4 flour was made by substituting 30% (v/v) wheat flour with RS4 (Fibersym RW, MGP Ingredients Inc., Atchison, KS). Chemical modification is used to produce this RS4 by cross-linking wheat starch with sodium trimetaphosphate. The process yields a phosphorylated cross-linked RS4 with 90% minimum dietary fiber content and with no more than 0.4% phosphorous. During the intervention participants exclusively used provided flours for all flour-based recipes. Flour-based food items were served at every meal, breakfast, lunch, and dinner. Centralized menu and meal preparations as well as uniform dining schedules and serving sizes within the colonies resulted in minimal interpersonal differences in feeding patterns among study participants resembling a fully controlled feeding. Even the colony members who did not participate in the study consumed the same foods and portion sizes due to the uniform menu and absence of alternatives. Dining outside of colonies is rare among the Hutterites. Thus, despite the intervention being conducted under free-living conditions and participants consuming their habitual diet, consistent dietary intakes were observed (29). Although many aspects of the Hutterite diet resemble typical low-in-sea-food continental Midwestern dietary patterns such as the use of baked goods, red meats, dairy, etc., one major difference is minimal use of processed, ready-to-eat, food items from supermarkets.

### Participants

An on-site screening and informed consent signing included a one-on-one interview on health history and medication usage with individual participants by trained research personnel. Inclusion criteria were age 18 years or older, both sexes, and willingness to comply with the research diet requirements, as well as sample and data collection protocols. Exclusion criteria included: pregnancy, lactation, long-term antibiotic therapy, immune-compromised, cancer, and other conditions that would affect the ability to provide informed consent or comply with the study protocol. Figure 1 summarizes the trial profile and number of participants in the parent trial as well as how 14 participants with metabolic syndrome were selected for this secondary analysis. The selected cohort included nine females and five males, aged 33–69 years with BMI ranging from 26.7 to 40.37 kg/m^2^. The presence of metabolic syndrome at baseline was determined based on the International Diabetes Federation criteria which we previously described (29).

### Biospecimen and data collection

Data (height, weight, waist, hip, fat and lean mass, diet record, medication usage) and biospecimens (stool and plasma) were collected on-site at baseline, 12 (end of first diet-phase), 14 (end of washout period), and 26 (end of second diet-phase) weeks. As appropriate, samples were immediately refrigerated or snap-frozen for transporting back to South Dakota State University’s Nutrigenomics Research Laboratory. Overnight fasted blood samples were collected by venipuncture from the antecubital vein in heparin-coated vacutainer tubes (BD Biosciences, Franklin Lakes, NJ). The biospecimens (both stool and plasma) were stored at −80°C until analyzed for downstream analyses. A self-administered semi-quantitative 3-day (2 weekdays and 1 weekend day) food frequency questionnaire featuring Hutterite food items was analyzed using Nutritionist Pro (Axxya Systems, Redmond, WA). A WebMD portion-size guide was printed and provided to participants for reference purposes.

### Chromatographic and mass spectrometric conditions for absolute quantification of bile acids

Plasma and fecal samples were shipped on dry ice to the NIH Metabolomics Center at UC-Davis (Davis, CA) and stored at −80°C upon arrival. For assaying, their published protocols for BA using an isotopically labeled surrogate standard mixture (glycocholic acid-d4, taurochenodeoxycholic acid-d4, cholic acid-d4, glycochenodeoxycholic acid-d4, chenodeoxycholic acid-d4, deoxycholic acid-d4, and Lithocholic acid-d5) and multiple reaction monitoring method in LC-MS/MS were used (35–38). BA were targeted using specific precursor and product ions, and the quantitative data was generated using calibrated standard curves for each BA.

#### Sample preparation

Plasma aliquots (50 μL/sample) were thawed on ice, briefly vortexed, and transferred to a polypropylene 96-well plate for extraction. The samples were spiked with 25 μL of a prior-optimized panel of isotope-labeled BA internal standards. The mixture was treated with 25 μL of antioxidant solution consisting of 0.2 mL/mL butylated hydroxytoluene and ethylene diamine tetra acetic acid. Next, 25 μL of 1,000 nanomolar (nM) 1-cyclohexyluriedo-3-dodecanoic acid and 1-phenyl 3-hexadecanoic acid urea were added to quantify surrogate standard recoveries which served as a quality control check for the extraction process. 125 μL of 1:1 v/v mixture of acetonitrile: methanol was added to a final volume of 250 μL. The samples were then vortexed for 30 min to precipitate protein and were centrifuged at 15,000 relative centrifugal force for 5 min. The resulting supernatant was then transferred to a 0.2 μm polyvinylidene fluoride membrane filter plate. The resulting solutions were stored at −20°C until analyzed in new polypropylene 96-well plates.

Stool samples (25 mg/sample) were thawed on ice. The samples were spiked with 20 μL of the isotope-labeled internal standard panel (for recovery quantification). The mixture was then treated with 10 μL of antioxidant solution of 1:5 butylated hydroxytoluene: ethylene diamine tetra acetic acid. Next, 500 μL of methanol was added as extraction solvent with stainless steel grinding balls followed by homogenization using GenoGrinder (SPEX SamplePrep, Metuchen, NJ) for 2 × 30 s at 1,350 rotation per minute. The homogenized mixture was centrifuged at 10,000 relative centrifugal force and the resulting supernatant was transferred to a 1.5 mL Eppendorf tube. The pellets were again subjected to the second round of homogenization to improve extraction efficiency. Recovered supernatants were then evaporated to dryness before reconstituting with 200 μL of 100 nM 1-cyclohexyluriedo-3-dodecanoic acid and 1-phenyl 3-hexadecanoic acid urea in 1:1 methanol/acetonitrile mixture. The reconstituted samples were again centrifuged at 10,000 relative centrifugal force for 3 min, the supernatant was transferred to polyvinylidene fluoride membrane filter plate and again centrifuged for 6 min. Finally, the resulting solutions were stored at −20°C polypropylene 96-well plates.

#### Instrument analysis and data processing

A total of 5 μL samples were injected onto Waters Acquity Ethylene Bridged Hybrid C18 column (1.7 μm particle size, 2.1 mm × 150 mm) in a Waters Acquity Ultra Performance Liquid Chromatography I-Class system (Waters Corporation, Milford, MA, USA) with an additional pre-column (1.7 μm particle size, 2.1 mm × 5 mm) with a column temperature at 45°C. The mobile phases consisted of LC-MS grade water with 0.1% formic acid for mobile phase (A) and acetonitrile with 0.1% formic acid for mobile phase (B). The 20 min gradient elution was carried out with a constant column flow of 400 μL/min. The spectral data were collected in negative electrospray ionization mode using scheduled multiple reaction monitoring on Sciex 4000 QTrap (AB Sciex, Framingham, MA). Multiple reaction monitoring was done utilizing optimized collision energies, de-clustering potentials, and collision cell exit potentials to target individual BA (more information in Supplementary Table 1). The targeted BA were then quantified against 6-point calibration curves using the internal standards. Peak integration, peak area computation, and quantification were carried out using MultiQuant 3.0 (AB Sciex, Framingham, MA). Finally, the average concentrations (nM) of two technical replicates were used for downstream analyses.

### Functional prediction from targeted metagenomics sequence

We have previously published the methodologies used for microbiota compositional analyses (8). Briefly, stool DNA was extracted using QIAamp DNA Stool Mini Kit (QIAGEN, Valencia, CA) following the manufacturer’s instructions. Samples were then quantified *via* the Qubit Quant-iT dsDNA Broad-Range Kit (Invitrogen, Life Technologies, Grand Island, NY). DNA samples were sent to Second Genome (South San Francisco, CA) for 16S V4 rRNA gene sequencing and operational taxonomic unit (OUT) identification (8). Sequencing was carried out on Illumina MiSeq for 250 cycles with paired-end sequencing. Multiplexed sequence reads were converted to taxonomic and phylogenetic profiles to construct an OTU table using QIIME and open reference OTU-picking against Greengenes reference database clustered at 97% by uclust (closed reference OTU picking). The longest sequence from each OTU was then assigned taxonomic classification *via* Mothur’s Bayesian classifier, trained against the Greengenes database clustered at 98%. Raw sequences are deposited in NCBI sequence read archive (SRA, accession number SRP035338), belonging to BioProject accession number PRJNA308315. Here, we used the existing 16s rRNA gene sequences to infer the functional potential of the microbial signatures detected in human stools from the representative orthologs using Piphillin algorithm (Second Genome Inc., South San Francisco, CA, USA) (39, 40). While no practical markers are available to quantify the metabolic functions of the gut microbiome, tools such as Piphillin can be used to predict the metabolic capacity of the microbial community by mapping the 16S rRNA gene sequences to known reference genomes. It utilizes sequence similarities as a measure of phylogenetic distances to predict metagenomic dynamics with respect to Kyoto Encyclopedia of Genes and Genomes (KEGG) orthologs (41, 42). Briefly, a raw OTU count table and the associated sequence data were uploaded to the publicly available Piphillin server using a 97% sequence identity cutoff to retrieve auto-generated data for downstream statistical analyses.

### Statistics and bioinformatics

#### General statistical considerations

Data analyses were performed in R version 4.1.0 (2021-05-18) and the data visualizations were carried out using R studio (packages: ggplot2, corrplot, ggcorrplot, ggheatmap, lattice, reshape2, ggpubr, Hmisc), MicrobiomeAnalyst (43), or MetaboAnalyst 5.0 (44) using our previously published workflow (8, 39, 40). We assessed the normality of the data using Shapiro-Wilk test, and when required, non-parametric transformations were carried out. Statistical significance was considered at *p* ≤ 0.05, while a *p*-value between 0.05 and 0.08 indicates approaching significance, when shown. All data are presented as either mean with standard deviation or as least-square means with standard error. Data analyses were performed using two approaches: effects of RS4 intervention were determined by comparing end-point data post-diet with respect to control, while effects of RS4 intervention alone were presented by comparing with baseline data. Statistical testing was done using linear mixed-effect models adjusted for weight, diet sequence, and inter-individual variations. The difference between microbiome functional capacity was evaluated using principal component analysis (PCA) and heatmap analysis with Euclidean distance matrix and Ward clustering algorithm, and the correlations were analyzed using Spearman’s rank-based method.

#### Bile acid analysis

BA that were detected below the limit of quantification value as mentioned in Supplementary Table 1 were excluded from statistical analyses. Individual BA concentrations were subjected to generalized log transformation for reducing skewness from the data before downstream analyses. Multivariate techniques used were: (i) partial least square-discriminant analysis (PLS-DA), a technique to identify metabolites that carry the greatest group-separating information derived from a weighted sum of the squared correlations between metabolites, represented as latent variables. Optimum number of components for the classification with PLS-DA was performed using 10-fold cross validation method with Metaboanalyst 5.0. (ii) Variable importance in projection (VIP), VIP scores estimate the relative importance of a variable in the PLS-DA model, derived from a weighted sum of the squared correlations between PLS-DA components and metabolites. The cut-off for the significant metabolite features was set as VIP scores > 1.0, (iii) heatmap analysis, to visualize the differences in abundance between individual BA, and (iv) PCA, as an unsupervised method of dimensionality reduction. The main purpose of multivariate techniques in the study is exploratory to identify varying BA between intervention and control and were not designed to be statistically predictive. Therefore, further univariate analyses were performed as mentioned above with linear mixed-effect model to identify statistical significance.

## Results

### Dietary intakes during interventions and participant characteristics

**FIGURE 2:**
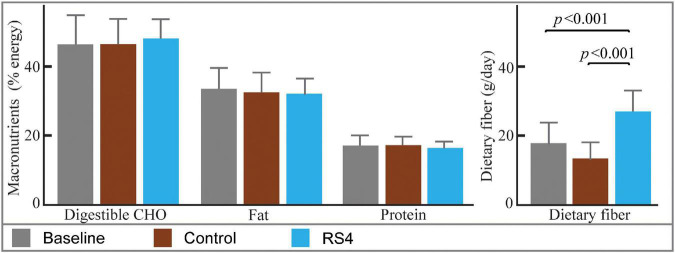
Estimated nutrient intakes at baseline and post diets. Bar graphs with mean ± SD showing similar macronutrient intakes at baseline and after interventions (all, *p* > 0.05). The difference in fiber intake was due to RS4 supplementation in the test group; *n* = 14/group; RS4, resistant starch type 4; CHO, Carbohydrates.

Differences in macronutrient intake can potentially influence the gut microbiota, confounding the effects of the intervention on BA (45, 46). Although meals were not provided by the researchers, the Hutterite cultural practices of communal kitchen and dining created a habitual-diet feeding model in a real-world setting closely matching a controlled-feeding approach—the gold standard for showing causality in free-living humans. As a result, digestible macronutrient intakes were similar between RS4 and control groups with ∼17, 46, and 33% of calories from protein, carbohydrate, and fat, respectively (all, *p* > 0.05 comparing the two interventions, Figure 2). The levels of macronutrient intake were within the Dietary Reference Intake ranges (47). Average energy intake per day in the selected sub cohort for this study (Figure 1) was similar between the intervention groups: 1,549 ± 511 kcal and 1,546 ± 431 kcal in RS4 and control, respectively. The similarity in total calories and macronutrients intake between the control and RS4 resulted in minimal differences in body weight, body mass index, waist circumference, fat and lean mass, and blood pressure, therefore, further reducing the confounding variables (Table 1). Due to the supplementation of RS4, there was a 12 g/day difference in fiber intake between the control and RS4 groups. RS4 content counted toward total dietary fiber since this specific food ingredient is on Food and Drug Administration’s approved dietary fiber list. Total daily fiber consumption in the RS4 group met the recommended fiber intake levels of the U.S. Dietary Guidelines (14 g/day per 1,000 calorie intake, DRI source: USDA, 2020-2025) (48). Fiber intake was significantly lower than recommended at baseline and in the control intervention group (both, *p* < 0.001, Figure 2).

**TABLE 1:** Anthropometric and clinical characteristics of the study participants at baseline and post-interventions.

Features	Baseline mean ± SD	Control mean ± SD	RS4 mean ± SD
Sex (M/F)	5/9	5/9	5/9
Age (years)	51.8 ± 11.2	51.8 ± 11.2	51.8 ± 11.2
Height (cm)	167.9 ± 8.9	167.9 ± 8.9	167.9 ± 8.9
Weight (kg)	91.6 ± 10.8	91.2 ± 11.3	91.1 ± 11.3
BMI (kg/m^2^)	32.6 ± 3.9	32.4 ± 3.9	32.4 ± 4.0
Waist (cm)	107.8 ± 10.0	107.4 ± 9.9	105.9 ± 9.9
Fat mass (kg)	33.5 ± 6.6	34.1 ± 6.1	33.8 ± 6.6
Lean mass (kg)	58.2 ± 12.1	58.1 ± 12.1	58.3 ± 12.3

Data are mean ± SD, *n* = 14; RS4, resistant starch type-4; M/F, male/female; BMI, body mass index; all *p* > 0.05, no significantly different features between the timepoints.

### Impact of resistant starch type 4 intake on overall plasma and stool bile acids (multivariate analyses)

**FIGURE 3:**
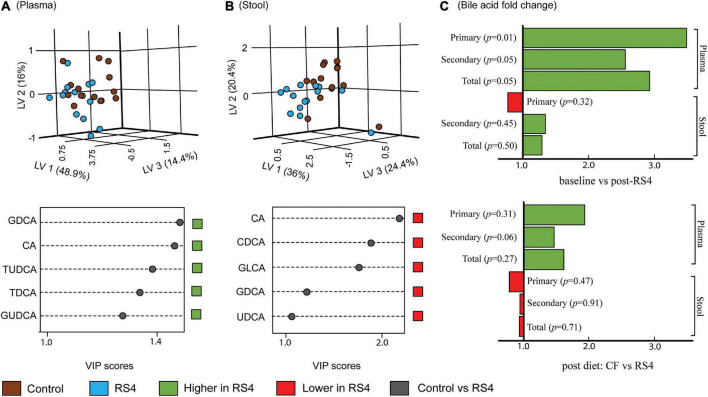
Effects of RS4 intervention on plasma and stool bile acids. Multivariate partial least squares-discriminant analysis (PLS-DA) score plots of control (brown) vs. RS4 (blue) in plasma **(A)** and stool **(B)** bile acids with their corresponding variable importance in projection (VIP) scores plots; Each dot represents the unique signature of 15 primary and secondary bile acid species for an individual; species with VIP > 1 are shown **(C)** bar graph showing fold change in bile acid group abundance; *p* calculated using linear mixed-effect model controlling for body weight, intervention sequence, and participant ID; *n* = 14/group; RS4, resistant starch type 4; LV, latent variable; CA, cholic acid; CDCA, chenodeoxycholic acid; DCA, deoxycholic acid; LCA, lithocholic acid; G, T, and U represent glyco-, tauro-, and urso-, respectively.

Multiple reaction monitoring-based high throughput metabolomics identified 23 BA species in plasma and stool samples collected at various time points. Fifteen of these 23 compounds met the pre-determined limit of quantification cut-off (more information in Supplementary Table 1) and were analyzed further using the PLS-DA model and subsequently also visualized as three-dimensional scores plots to reveal metabolites that carry the greatest group-separating information, represented as latent variables between control and RS4 groups (Figures 3A,B). The three-dimensional PLS-DA explained a total of 79.3% (x, 48.9%; y, 16%; and z, 14.4%) and 80.8% (x, 36%; y, 20.4%; and z, 24.4%) variations in plasma and stool BA characteristics, respectively, between the intervention groups. Applying a > 1 VIP score cutoff, we identified five BA species that contributed maximally to the observed variations in overall stool and plasma BA profiles, respectively, distinguishing RS4 from the control intervention. These species were: glycodeoxycholic acid, cholic acid, tauroursodeoxycholic acid, taurodeoxycholic acid, and glycoursodeoxycholic acid that increased in plasma post-RS4; and cholic acid, chenodeoxycholic acid, glycolithocholic acid, glycodeoxycholic acid, and ursodeoxycholic acid in stool that decreased after RS4 intake. When the BA species were grouped as total, primary, and secondary for each of stool and plasma and analyzed using linear mixed-effect modeling, an increase in primary, secondary, and total BA from baseline to post-RS4 in plasma was observed (all, *p* ≤ 0.05), whereas, similar shifts were not determined in the stool (all, *p* > 0.1) (Figure 3C). In addition, increase in total secondary BA in plasma was approaching significance in the RS4 group relative to the control (*p* = 0.06). We speculated that individual BA species may have contributed to the observed overall changes in plasma BA, which was further examined using univariate analyses.

### Impact of resistant starch type 4 intake on circulating bile acid species (univariate analyses)

**FIGURE 4:**
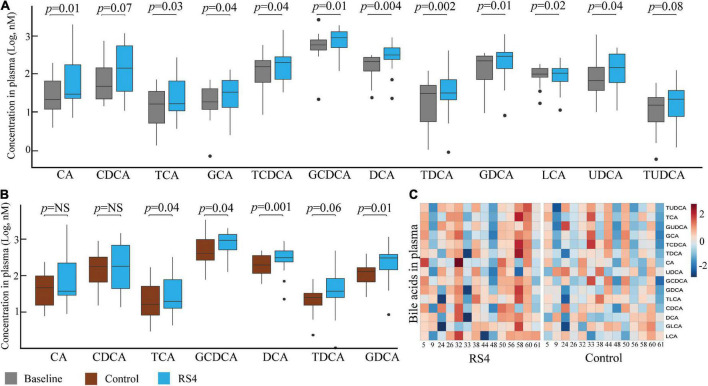
RS4 altered secondary bile acids. Box plots comparing concentrations of individual primary, secondary, and conjugated bile acid species: at baseline and after RS4 intake **(A)** after control vs. RS4 interventions **(B)**; for **(A,B)** *p*-values were calculated using linear mixed-effect model controlling for body weight, intervention sequence, and participant ID; lower and upper hinges of the boxplot denote 25th and 75th percentile, line denotes median, and whiskers are drawn to minimum and maximum values but not further than 1.5x interquartile range. Outliers are displayed as black dots; **(C)** heatmap showing individual study participants on x-axis and bile acid species on y-axis to present differential abundance of circulating bile acids between individuals and groups; the colored cells denote abundance (red- high abundance, blue- low abundance); variables were autoscaled; For all panels: *n* = 14/group; RS4, resistant starch type 4; CA, cholic acid; CDCA, chenodeoxycholic acid; DCA, deoxycholic acid; LCA, lithocholic acid; G, T and U represent glyco-, tauro-, and urso-, respectively.

Linear mixed-effect modeling analyses were performed individually for each of the 15 circulating BA species. Glycochenodeoxycholic acid (≥ 33%, highest abundance), deoxycholic acid, ursodeoxycholic acid, chenodeoxycholic acid, taurochenodeoxycholic acid, and glycodeoxycholic acid were the six most abundant BA at all-time points. As for within-group changes, the circulating concentrations for these six BA increased from baseline to post-RS4: 452–1,183 nM (glycochenodeoxycholic acid, *p* = 0.01), 170–546 nM (deoxycholic acid, *p* = 0.004), 145–260 nM (ursodeoxycholic acid, *p* = 0.04), 143–280 nM (chenodeoxycholic acid, *p* = 0.07), 98–210 nM (taurochenodeoxycholic acid, *p* = 0.04), and 77–263 nM (glycodeoxycholic acid, *p* = 0.01) (Figure 4A). These individual increases from baseline also contributed to an increase in total circulating BA post RS4 (1,108–3,072 nM, *p* = 0.057). Of note, 10 out of 15 BA were significantly enriched with the RS4 diet, while none of those same species were modulated in the control diet group (Figure 4A).

Comparing between the intervention groups, RS4 group had higher plasma concentration of total BA and several individual species with respect to the control group: total (3,072 nM vs. 2,275 nM, *p* = 0.057), taurocholic acid (41 nM vs. 21 nM, *p* = 0.042), taurodeoxycholic acid (49 nM vs. 31 nM, *p* = 0.058), glycochenodeoxycholic acid (1,183 nM vs. 748 nM, *p* = 0.039), glycodeoxycholic acid (263 nM vs. 147 nM, *p* = 0.009), and deoxycholic acid (546 nM vs. 309 nM, *p* = 0.001) (Figures 4B,C). These species belong to the conjugated or secondary (microbiota-dependent) BA classes. A similar trend of higher circulating primary BA concentration (cholic acid and chenodeoxycholic acid) was not observed in the RS4 group (Figure 4B). Thus, we speculate that the impact of RS4 on BA-metabolism may be primarily directed through its modulation of the gut microbiota.

### Association of the microbiota with secondary bile acids

**FIGURE 5:**
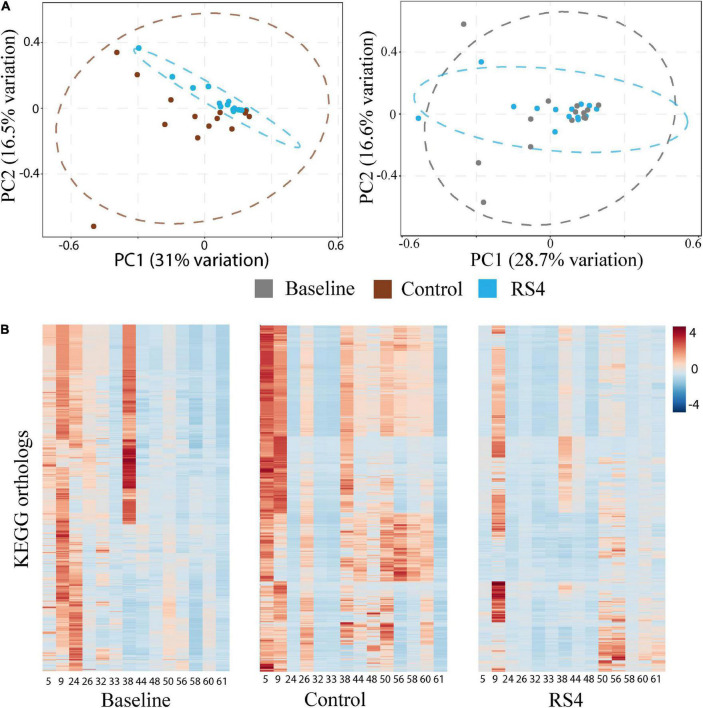
Impact of RS4 on gut microbiota functional capability. **(A)** Principal component analysis (PCA) of KEGG orthologs based on 16s rRNA gene sequences showing higher variability in control as illustrated by wider 95% CI (elliptical boundaries) vs. RS4 intervention that clustered the predicted orthologs toward zero on both axes within a narrow spatial area; the x and y axes explain 31 and 16.5% variability, respectively, in the control vs. RS4 the distinction between diets was less evident with baseline vs. RS4; each dot represents a unique signature of an individual participant’s gut microbiota for the 8,621 KEGG orthologs; dataset was log transformed. **(B)** Heatmap showing individual study participants on x-axis and KEGG orthologs on y-axis to present differential abundance of orthologs between individuals and groups; the colored cells denote abundance (red- high abundance, blue- low abundance); variables were autoscaled; and *n* = 14/group; RS4, resistant starch type 4; KEGG, Kyoto encyclopedia of genes and genomes.

The potential role of microbial species to upregulate secondary BA was explored using Spearman’s rank-based test. The gut microbiota species that our group previously reported (8) as altered after RS4 consumption were re-assessed for the possible associations with the secondary BA in this sub-cohort. *Bifidobacterium adolescentis* showed RS4 specific association, with multiple secondary BA species (Table 2): deoxycholic acid (rho = 0.55, *p* = 0.05), glycodeoxycholic acid (rho = 0.65, *p* = 0.02), and taurodeoxycholic acid (rho = 0.56, *p* = 0.04), whereas, similar correlation was not observed after control intervention (all, rho < 0.5 and *p* > 0.1). We also used a prediction analysis of KEGG ortholog-based functional capability associated with the microbiota differences between control and RS4 groups. Principal component analyses demonstrated separation and overlap of 8,621 unique KEGG orthologs based on cluster predictions (Figure 5A). However, the same crossed-over participants in the control intervention group did not produce similar tight clustering; this is illustrated by the dotted ellipse showing a substantially wider 95% confidence interval. This also shows that the range of inter-individual variability in microbiome functional output was much greater after control flour intake compared to RS4 consumption. A heatmap analysis further supports between-group variations (Figure 5B), showing differential up or downregulations of KEGG orthologs between the groups to indicate RS4-specific modulation of the gut microbiota.

**TABLE 2:** RS4 specific correlation of *Bifidobacterium adolescentis* with secondary bile acids in plasma.

Bile acids	Control flour		Resistant starch 4	
		
	rho	*P*-value	rho	*P*-value
**Deoxycholic acid**	0.23	0.46	0.55	0.05
**Glycodeoxycholic acid**	0.46	0.12	0.65	0.02
**Taurodeoxycholic acid**	0.43	0.14	0.56	0.04

Presented data calculated using Spearman’s rank correlation method. *n* = 14/group.

## Discussion

The study of diet-induced and microbiota-mediated metabolites helps better understand and when necessary, intervene the host-microbiota interactions in the context of health and diseases. It has increasingly become clear that BA are not only representative of host-microbiota interactions, but are also major regulators of metabolic processes in addition to their digestive functions. We believe that this human subject study enabled us to address knowledge gaps related to the impact of dietary RS4—with a range of previously demonstrated prebiotic and metabolic health effects—on BA. Our results show that circulating secondary and conjugated BA features were altered after RS4 intake in individuals with metabolic syndrome. Furthermore, *B. adolescentis* had an RS4 specific association with certain BA species—deoxycholic acid (secondary species), and its conjugates (glycodeoxycholic acid and taurodeoxycholic acid). Results also indicate that regular RS4 consumption influenced the functional capability of the gut microbiota.

We are not aware of an existing study reporting effects of RS4 on human circulating BA to compare our data with. However, a whole grain diet compared to a refined grain diet was shown to increase multiple human circulating BA species (49). Increased circulating deoxycholic acid reported in mice after RS2 intervention also aligns with our observation (28). Future research providing independent validation will be critical to contextualize previously reported metabolic health benefits of RS4 since changes in BA composition and pool size were proposed to be clinically relevant for healthcare applications (50, 51).

We note that the biological activities of individual BA species in the context of metabolic diseases remain poorly understood at this time due to inadequate research and a lack of scientific consensus. While some primary BA species have been linked with inflammation, insulin resistance and diabetes risk (49, 52–54), in mice, deoxycholic acid was shown to reduce inflammation, postprandial triglycerides, and cholesterol levels (55, 56). On the other hand a few observational data that remains unverified in clinical trials indicate cancer-promoting effect of deoxycholic acid (57, 58). Interestingly, synthetic deoxycholic, and ursodeoxycholic acids are approved pharmaceuticals to treat certain human ailments such as primary biliary cirrhosis, gallstones, hepatobiliary disorders, and reduction of subcutaneous fat deposits (59).

Our results indicate that RS4 consumption resulted in a higher circulating BA, especially the secondary as well as tauro- and glyco- conjugated species. The fecal excretion following consumption of RS4 was minimal or unchanged. This potentially indicates alteration of BA reabsorption in the intestine. Chiang et al. described that conjugation of BA species increases *in vitro* ionization and solubility at the physiological pH, improving rate of active transport in the intestinal brush border (3). Therefore, it is possible that modulation of enzymes involved in conjugation and deconjugation can potentially result in the elevation of circulating conjugated BA. This may also include microbial alteration of enzymes involved in secondary BA production. Notably, RS4 modulated the gut microbiota as well as the secondary BA pool size. Thus, the hypothesis is supported by our observation of the RS4-specific association between *B. adolescentis* and DCA and its conjugates. As Martínez et al. (9) and we have previously (8) reported, prebiotic effects of RS4 included enrichment of *B. adolescentis*, a species that is currently marketed as probiotics. Bifidobacterial species including *B. adolescentis* possess carbohydrate active enzymes which makes them well adapted for plant-based carbohydrate utilization (60, 61). *B. adolescentis* also shows the strain-specific immunomodulatory activity as well as the ability to feed other gut bacteria using RS as a prebiotic substrate (60). It is also speculated that Bifidobacterium directly or indirectly influence BA metabolism by altering bile salt hydrolase activity (62, 63). Future prospective studies may help elaborate RS4-mediated modulation of this species in the context of BA metabolism.

Experimental research has strengths and limitations. This exploratory study benefits from the robust double-blinded, placebo-controlled, crossover design of the parent trial that allowed for matching by age, sex, and other variability in participant features between the intervention groups. Unlike pharmaceutical trials, defining placebo-control and accomplishing double-blinding of dietary interventions are less common in nutrition research. Since all participants must consume food, identifying an appropriate placebo and then masking any organoleptic differences for the participants is not always possible, frequently confounding outcomes. The stealth properties of the RS4 ingredient used in the study and availability of the parent wheat flour used for crosslinked RS4 production made a double-blinded, placebo-controlled design feasible. In addition, the 12-week intervention in free-living, yet socio-economically homogenous participants who lived in small communities and dined together, potentially minimized confounding due to environmental variations in the gut microbiome. It is possible that these robust design-related features contributed to the identification of differential BA changes in the RS4 group despite a smaller sample size that was not specifically power-estimated for this exploratory analysis. Multiple reaction monitoring-based assay design improves repeatability, increases linear dynamic range, and avoids inaccurate peak quantification or false-positive peak detections. Furthermore, the targeted approach for metabolite identification and quantification using isotope-labeled internal standard-based calibration curves for individual BA species allowed generation of robust data. Given the lack of intervention studies assessing dietary impacts on human BA at this time, the absolute concentrations reported in this study can also be compared with future studies to establish clinical relevance. We noted that our study population was overweight or obese with metabolic syndrome features and although of European descent, belonged to a cultural-minority group. This specific population may have a different BA metabolism compared to healthy participants. Thus, caution is necessary for generalizing the findings to other populations. Future evaluations are warranted in larger cohorts with a range of clinical and anthropometric phenotypes.

## Conclusion

In conclusion, RS4 is a prebiotic food ingredient with metabolic health benefits as well as has stealth properties that can potentially mitigate dietary compliance issues faced by chronic health promotion programs. Despite an established role of the microbiota in BA metabolism and known ability of RS4 to reform microbiota, knowledge about the effects of RS4 on BA remained limited, especially in humans. The results from this hypothesis-generating study indicate that RS4 supplementation in the diet increases fiber consumption and suggests for the first time that RS4 modulates circulating BA in humans. Given the role of BA signaling in metabolic functions, future studies may reveal if BA alterations by RS4 contribute to its previously reported cholesterol-lowering and other metabolic health effects.

## Data availability statement

The datasets presented in this study can be found in online repositories. The names of the repository/repositories and accession number(s) can be found below: https://www.ncbi.nlm.nih.gov/, SRA, #SRP035338, BioProject #PRJNA308315.

## Ethics statement

The studies involving human participants was reviewed and approved by the Institutional Review Board for Human Subject Research at the South Dakota State University (approval number: 1112012-CR). The trial was registered at clinicaltrials.gov as NCT01887964. The patients/participants provided their written informed consent to participate in this study.

## Author contributions

MD: conceptualization, project administration, funding acquisition, methodology and data collection, manuscript editing, and final approval. SD: formal data curation and analyses, manuscript writing, and final approval. Both authors contributed to the article and approved the submitted version.

## Abbreviations

BA: Bile Acid
KEGG: Kyoto Encyclopedia of Genes and Genomes
LC-MS: Liquid Chromatography-Mass spectroscopy
OTU: Operational Taxonomic Unit
PLS-DA: Partial Least Square Discriminant Analysis
PCA: Principal Component Analysis
RS (RS4): Resistant Starch (type 4)
VIP: Variable Importance in Projection.
