# Supplementary material for: Resistant starch type-4 intake alters circulating bile acids in human subjects
Source: Front Nutr. 2022 Oct 20;9:930414. doi: 10.3389/fnut.2022.930414 (PMC9631925; doi:10.3389/fnut.2022.930414)
Supplement: Supplementary file 1 [file Table_1.DOCX]

**Resistant starch type-4 intake alters circulating bile acids in human subjects**

Samitinjaya Dhakal and Moul Dey*

School of Health and Consumer Sciences, Box 2275A, South Dakota State University, Brookings, SD 57007

*Correspondence to Moul.Dey@sdstate.edu; 605-688-4050.

| **Bile Acids** | **MRM** | **RT** | **DP** | **CE** | **EP** | **CXP** | **LOQ (nM)** |
| --- | --- | --- | --- | --- | --- | --- | --- |
| **Primary Bile Acids** |  |  |  |  |  |  |  |
| Cholic acid | 407.3 / 407.3 | 10.69 | -115 | -30 | -10 | -9 | 10 |
| Chenodeoxycholic acid | 391.3/ 391.3 | 12.57 | -105 | -30 | -10 | -9 | 10 |
| Taurocholic acid | 514.3 / 80 | 6.47 | -155 | -110 | -10 | -4 | 10 |
| Glycocholic acid | 464.3 / 74 | 8.01 | -110 | -70 | -10 | -10 | 5 |
| Glycochenodeoxycholic acid | 448.3/ 74 | 11.32 | -115 | -70 | -10 | -4 | 2 |
| Taurochenodeoxycholic acid | 498.3 / 80 | 8.87 | -145 | -110 | -10 | -4 | 2 |
| **Secondary Bile Acids** |  |  |  |  |  |  |  |
| Tauro-ω-Muricholic acid* | 514.3 / 80 | 3.53 | -155 | -110 | -10 | -4 | 10 |
| Tauro-α-Muricholic acid* | 514.3 / 80 | 3.77 | -155 | -110 | -10 | -4 | 10 |
| Tauro-β-Muricholic acid* | 514.3 / 80 | 3.92 | -155 | -110 | -10 | -4 | 10 |
| Tauroursodeoxycholic acid | 498.3 / 80 | 6.15 | -145 | -110 | -10 | -4 | 10 |
| ω-Muricholic acid* | 407.3 / 407.3 | 7.69 | -115 | -30 | -10 | -9 | 10 |
| Glycoursodeoxycholic acid | 448.3/ 74 | 7.81 | -115 | -70 | -10 | -4 | 2 |
| Glycohyodeoxycholic acid* | 448.3 / 74 | 7.98 | -115 | -70 | -10 | -4 | 5 |
| α-Muricholic acid* | 407.3 / 407.3 | 8.04 | -115 | -30 | -10 | -9 | 5 |
| β-Muricholic acid* | 407.3 / 407.3 | 8.43 | -115 | -30 | -10 | -9 | 10 |
| Taurodeoxycholic acid | 498.3 / 80 | 9.55 | -155 | -110 | -10 | -2 | 10 |
| Ursodeoxycholic acid | 391.3 / 391.3 | 10.94 | -105 | -30 | -10 | -9 | 5 |
| Glycodeoxycholic acid | 448.3 / 74 | 11.95 | -115 | -70 | -10 | -4 | 2 |
| Taurolithocholic acid | 482.3 / 80 | 12.32 | -150 | -110 | -10 | -4 | 5 |
| Deoxycholic acid | 391.3 / 391.3 | 12.65 | -105 | -30 | -10 | -9 | 10 |
| Glycolithocholic acid | 432.3 / 74 | 12.71 | -120 | -65 | -10 | -4 | 10 |
| Lithocholic acid | 375.3 / 375.3 | 13.15 | -130 | -35 | -10 | -8 | 10 |

**Supplementary table 1**: Multiple reaction monitoring transitions for individual bile acids

MRM: multiple reaction monitoring; RT: retention time; DP: declustering potentials; CE: collision energy; EP: entrance potential; CXP: collision cell exit potential; LOQ: limit of quantification; *Bile acids that did not meet minimum LOQ cut-off
